# Impact of socioeconomic deprivation on the development of diabetic retinopathy: a population-based, cross-sectional and longitudinal study over 12 years

**DOI:** 10.1136/bmjopen-2014-007290

**Published:** 2015-04-15

**Authors:** Liying Low, Jonathan P Law, James Hodson, Ritchie McAlpine, Una O'Colmain, Caroline MacEwen

**Affiliations:** 1 Academic Unit of Ophthalmology, University of Birmingham, Birmingham and Midland Eye Centre, Birmingham, UK; 2 Ninewells Hospital and Medical School, Dundee, UK; 3 University Hospitals Birmingham NHS Foundation Trust, Queen Elizabeth Hospital Birmingham, Birmingham, UK; 4 Tayside Diabetes Managed Clinical Network (MCN), Ninewells Hospital and Medical School, Dundee, UK; 5 Department of Ophthalmology, Ninewells Hospital and Medical School, Dundee, UK

**Keywords:** EPIDEMIOLOGY, SOCIAL MEDICINE

## Abstract

**Objective:**

To study the association between socioeconomic deprivation and prevalence of diabetic retinopathy (DR).

**Design:**

Population-based, cross-sectional observational study and retrospective longitudinal analysis over 12 years.

**Setting:**

Primary care, East of Scotland.

**Methods:**

Outcome data from DR screening examinations (digital retinal photography) were collected from the Scottish regional diabetes electronic record from inception of database to December 2012. The overall Scottish Index of Multiple Deprivation (SIMD) 2012 score for each patient was obtained using their residential postcode. Multiple binary logistic regression was used to analyse the relationship between overall SIMD score and prevalence of DR, adjusting for other variables: age, gender, glycated haemoglobin, cholesterol levels and duration of disease.

**Primary outcome:**

Any retinopathy (R1 and above) in either eye.

**Results:**

A total of 1861 patients with type 1 diabetes mellitus (DM) and 18 197 patients with type 2 DM were included in the study. Prevalence of DR in type 1 and type 2 DM were 56.3% and 25.5%, respectively. Increased prevalence of DR in type 1 DM was associated with higher overall SIMD score (p=0.002), with an OR for the most deprived relative to the least deprived of 2.40 (95% CI 1.36 to 4.27). In type 2 DM, the overall SIMD score was not significantly associated with increased prevalence of DR, with an OR for the most deprived relative to the least deprived of 0.85 (95% CI 0.71 to 1.02, p=0.07).

**Conclusions:**

Socioeconomic deprivation is associated with increased prevalence of DR in patients with type 1 DM and this occurs earlier. This highlights the need for targeted interventions to address inequalities in eye healthcare.

**Strengths and limitations of this study**

- Large sample size of 20 058 patients.
- Longitudinal cohort of patients followed up systematically using the validated Scottish Diabetic Retinopathy Screening protocol over 12 years.
- We have taken into account other potential confounders, such as duration of disease, glycated haemoglobin levels, cholesterol levels and blood pressure reading, into our analyses.
- Unable to account for mortality bias and time at risk for these patients.
- Unable to attain information on the changes in postcode of these patients.

## Introduction

Despite well-established national diabetes screening programmes, diabetic retinopathy (DR) and maculopathy remain major causes of visual impairment among the working-age population in the UK.^1^ Early detection through screening and prompt treatment may prevent progression to blindness,^2^ ^3^ thereby, justifying the provision of an equal and free-access retinopathy screening programme for all patients with diabetes mellitus (DM). In the year 2011/2012, an estimated £2.6 million was spent on DR screening in the UK.^4^ Undoubtedly, the high expenditure involved in running these screening programmes raises questions as to whether this is a wise investment and if inequalities truly exist in a free-access healthcare system such as the National Health Service (NHS).^5^

Previous large epidemiological studies evaluating the incidence of DR in patients with a poor socioeconomic background have been unable to account for major risk factors in the development of DR, such as poor glycaemic control and longer duration of disease, which may be more prevalent in patients in lower socioeconomic groups. Moreover, uptake of the screening programme is especially low among those living in deprived areas where access to the nearest eye care provider is limited.^6^ ^7^

Therefore, we seek to explore the association between socioeconomic deprivation and prevalence of DR in the East of Scotland where there is a well-established Scottish Diabetic Retinopathy Screening programme, adjusting for the known risk factors of DR: duration of disease, glycated haemoglobin (HbA1c) levels, blood pressure (BP) and cholesterol levels.

## Methods

### Study population

All patients with DM above the age of 12 years and registered with a general practice in Tayside were referred to the Scottish Diabetic Retinopathy Screening Service, excluding those who were unable to attend screening due to infirmity and those already attending the hospital ophthalmology services. We included all patients with type 1 and type 2 DM who attended DR screening in Tayside from inception of database until December 2012. For the longitudinal analysis, we considered only those patients who received a diagnosis of DM from year 2000 onwards and did not have retinopathy at time of diagnosis. Patients were followed up until December 2012.

### The Scottish Care Information Diabetes Collaboration system

The Scottish Care Information Diabetes Collaboration (SCI-DC) data set holds information on the demographic characteristics of each patient diagnosed with DM, such as the postcode of residence, registration of general practitioner (GP), year of birth and date of diagnosis of DM. The SCI-DC data set also includes clinical parameters, which are measured at least once a year after the diagnosis of diabetes: HbA1c, cholesterol levels, systolic blood pressure (sBP) and diastolic blood pressure (dBP). We obtained the most recent DR status, HbA1c, cholesterol, sBP and dBP levels for this study.

### Socioeconomic deprivation

We used the Scottish Index of Multiple Deprivation (SIMD) 2012 score as a measure of area-based deprivation.^8^ The SIMD score for each geographical data zone is a combination of 38 indicators of deprivation across seven broad domains: education, crime, geographic access, income, skills and training employment, health, and housing. We used the residential postcodes to assign each patient an individual SIMD 2012 score. The higher the SIMD 2012 score, the more deprived the area of residence. The SIMD score is used to group the data zones into quintiles: 0–20% most deprived, 20–40%, 40–60%, 60–80%, 80–100% least deprived.

### Scottish Diabetic Retinopathy Screening protocol

The protocol for Scottish Diabetic Retinopathy Screening has been widely published.^9^ To summarise, all patients with diabetes in the East of Scotland undergo free annual DR screening; this is provided by two mobile units equipped with digital fundus cameras which travel to every GP practice, and a static camera site in Dundee.^6^ Grading of DR and maculopathy is performed from digital retinal photographs and outcomes stored in the regional screening database linked to the SCI-DC. The use of digital retinal photographs for DR screening in Scotland has been previously validated.^10^ ^11^

The primary outcome of the study was presence of background DR (R1) or worse, in either eye.

### Statistical analyses

Initially, cross-sectional analyses were performed to assess the relationship between a range of factors and the presence of DR. This took the form of multivariable binary logistic regression models, with the dependent variable indicating whether the most recent screening for each patient identified DR (R1 or worse) in either eye. The SIMD score was included as the covariate, along with a range of other potentially confounding factors, such as duration of disease, HbA1c levels, BP and cholesterol levels. The shape of the relationships between continuous covariates and the outcome were assessed prior to the analysis, with log_10_ transformations applied, where necessary, to ensure good model fit. In order to test for selection bias within patients enrolled in the diabetes screening programme (SCI-DC database), comparisons were made between those who did not attend any retinopathy screening and those who were screened at least once, using the Mann-Whitney test.

A secondary, longitudinal analysis was then performed, considering the average time that patients with DM took to develop retinopathy. As SIMD data were only available for patients in 2012, we only considered those patients diagnosed with diabetes from the year 2000 onwards, in order to minimise the potential for variability over time in the factors being considered. During the period of follow-up for each patient, the results of every screening test were reviewed. The ‘time to event’ was calculated as the time from diagnosis to the first test showing retinopathy, with patients being censored at the end of the period if they did not develop retinopathy. The data were then analysed using the Kaplan-Meier approach, with separate curves for each quintile of the SIMD score, which were compared using log-rank (Mantel-Cox) tests. In addition to this, a Cox regression model was produced, which accounted for patients’ age and gender as additional risk factors.

For all of the analyses, patients with type 1 and type 2 DM were treated separately. All analyses were performed using IBM SPSS V.19 (IBM Corp. Armonk, New York, USA). Missing data were excluded on a per-analysis basis and p<0.05 was deemed to be indicative of statistical significance.

## Results

A total of 1861 patients with type 1 DM and 18 197 patients with type 2 DM were included in the study. The baseline demographic data are summarised in table 1. Prevalence of DR in type 1 and type 2 DM were 56.3% and 25.5%, respectively. There was no statistically significant difference in the SIMD scores between the attenders and non-attenders to screening (table 2).

**Table 1:** Baseline demographic data

	Type of diabetes		
	Missing (%)	Type 1	Type 2
N		1861	18 197
Age*	–	40.6 (17.8)	67.1 (12.5)
Sex	–		
Male		1017 (54.7%)	9896 (54.4%)
Female		844 (45.4%)	8301 (45.6%)
Duration (years)†	0.1	16.4 (7.40, 28.3)	6.52 (2.93, 11.3)
HbA1c† (mmol/mol)	1.2	74.0 (62.0, 87.0)	54.0 (48.0, 65.0)
Cholesterol value (mmol/L)*	1.0	4.58 (1.28)	4.25 (1.05)
Systolic BP (mm Hg)*	0.9	131.0 (16.3)	134.8 (15.7)
Diastolic BP*	0.9	74.4 (10.1)	74.7 (10.1)
Overall SIMD 2012 score†	1.0	14.1 (9.78, 28.0)	14.8 (9.91, 29.0)
Health†		−0.18 (−0.58, 0.43)	−0.87 (−0.51, 0.50)
Education skills and training†		−0.14 (−0.63, 0.54)	−0.08 (−0.64, 0.60)
Housing†		15.1 (9.66, 25.2)	15.8 (10.1, 26.4)
Geographic access†		16.3 (7.2, 30.8)	15.0 (6.15, 27.9)
Crime†		260.0 (140.0, 543.0)	284.0 (148.0, 548.0)
Retinopathy	5.6	1733	17 226
No retinopathy		758 (43.7%)	13 184 (76.5%)
With retinopathy		975 (56.3%)	4042 (23.5%)
BDR mild		794 (45.8%)	3656 (21.2%)
BDR observable		63 (3.6%)	208 (1.2%)
BDR referable		54 (3.1%)	104 (0.6%)
Proliferative retinopathy		64 (3.7%)	74 (0.4%)
Maculopathy	8.9	1619	16 667
No maculopathy		1146 (70.8%)	15 479 (92.9%)
With maculopathy		473 (29.2%)	1188 (7.1%)
Observable maculopathy		91 (5.6%)	165 (1.0%)
Referable maculopathy		382 (23.6%)	1023 (6.1%)

*Data presented as mean (SD).

†Data presented as median (25th and 75th quartile).

BDR, background diabetic retinopathy; BP, blood pressure; HbA1c, glycated haemoglobin; SIMD, Scottish Index of Multiple Deprivation.

**Table 2:** SIMD scores by attendance

	Attenders		Non-attenders		
DM	N (%)	SIMD	N (%)	SIMD	p Value
Type 1	1720 (93.3%)	14.16 (9.78–28.10)	124 (6.7%)	14.10 (9.79–23.94)	0.665
Type 2	17 081 (94.8%)	14.81 (9.87–29.04)	941 (5.2%)	15.00 (10.04–30.29)	0.101

SIMD data reported as: ‘median (quartiles)’, with p values from Mann-Whitney tests. ‘Non-attenders’ are those that have never been tested for retinopathy.

DM, diabetes mellitus; SIMD, Scottish Index of Multiple Deprivation.

Multivariable analysis (table 3) found strong associations in both types of DM between the development of retinopathy and HbA1c level, BP, duration of disease, gender and cholesterol levels. After accounting for these effects, increasing SIMD score was found to be associated with an increased prevalence of DR in patients with type 1 DM (OR for a 10 unit increase in score: 1.13, 95% CI 1.04 to 1.22, p=0.002). Since the SIMD score in our cohort ranges from 2 to 75, this is equivalent to an OR of 2.40 (95% CI 1.36 to 4.27) for patients from the most deprived areas relative to the least deprived. This effect was not observed in patients with type 2 diabetes with the OR for the most deprived relative to the least deprived being non-significant at 0.85 (95% CI 0.71 to 1.02, p=0.074). The multivariable analysis was also repeated including only the non-modifiable confounding factors (ie, age, gender and disease duration), which returned comparable results.

**Table 3:** Multivariate analysis of factors associated with diabetic retinopathy

	Type 1 DM		Type 2 DM	
	OR (95% CI)	p Value	OR (95% CI)	p Value
Gender (male)	1.38 (1.09 to 1.75)	0.006*	1.19 (1.11 to 1.29)	<0.001*
Age (decades)	0.92 (0.84 to 0.99)	0.036*	0.90 (0.86 to 0.93)	<0.001*
Disease duration (decades)	35.0† (22.5 to 54.5)	<0.001*	2.89 (2.72 to 3.07)	<0.001*
HbA1c (×10 mmol/mol)	1.17 (1.10 to 1.24)	<0.001*	1.12 (1.10 to 1.15)	<0.001*
Cholesterol	1.14 (1.01 to 1.29)	0.029*	0.94 (0.90 to 0.97)	0.001*
Systolic BP (×10 mm Hg)	1.03 (0.95 to 1.13)	0.479	1.11 (1.08 to 1.14)	<0.001*
Diastolic BP (×10 mm Hg)	1.16 (1.02 to 1.33)	0.024*	0.95 (0.91 to 0.99)	0.028*
Overall SIMD score (×10)	1.13 (1.04 to 1.22)	0.002*	0.98 (0.95 to 1.00)	0.074

*Significant at p<0.05.

†Variable was log_10_-transformed prior to analysis, so the coefficient represents the OR for a 10-fold increase in disease duration.

BP, blood pressure; DM, diabetes mellitus; HbA1c, glycated haemoglobin; SIMD, Scottish Index of Multiple Deprivation.

**Figure 1:**
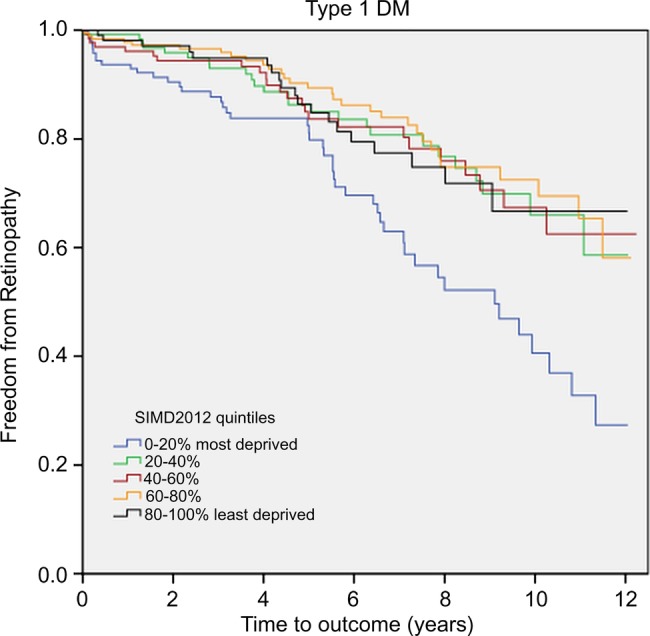
Kaplan-Meier survival curve of freedom from retinopathy in patients with type 1 DM. DM, diabetes mellitus; SIMD, Scottish Index of Multiple Deprivation.

The median time from diagnosis of DM to onset of retinopathy (R1) in patients with type 1 DM from the most deprived areas was 9.1 years as opposed to more than 12 years in all other quintiles (p<0.001; figure 1). The 10 years retinopathy-free survival for patients with type 1 DM in the most deprived areas was 40.6% compared with 66.7% for patients from the least deprived areas. A Cox regression model, accounting for age and gender, returned a HR of 2.16 (95% CI 1.27 to 3.69) for the most deprived, relative to the least deprived quintile (p<0.001). For the remainder of the deprivation quintiles, retinopathy hazards were similar. Hence, the increased risk of retinopathy appears to be mainly confined to the 0–20% most deprived group of patients (table 4).

**Table 4:** Retinopathy-free survival rates and HRs for type 1 DM according to the SIMD quintiles

		Survival (retinopathy-free)			
SIMD quintiles (%)	(N)	1 year (%)	5 years (%)	10 years (%)	HR (95% CI)*
0–20 most deprived	145	93.7	82.5	40.6	2.16 (1.27 to 3.69)
20–40	134	99.3	86.3	66.0	0.95 (0.52 to 1.74)
40–60	130	96.1	83.7	67.3	0.94 (0.51 to 1.72)
60–80	188	97.8	89.4	72.5	0.80 (0.45 to 1.43)
80–100 least deprived	112	98.1	86.4	66.7	–

*ORs are derived from a Cox regression model, which also accounts for age and gender, and are relative to the most deprived SIMD quintile. SIMD quintile was significant in this model (p<0.001).

DM, diabetes mellitus; SIMD, Scottish Index of Multiple Deprivation.

**Figure 2:**
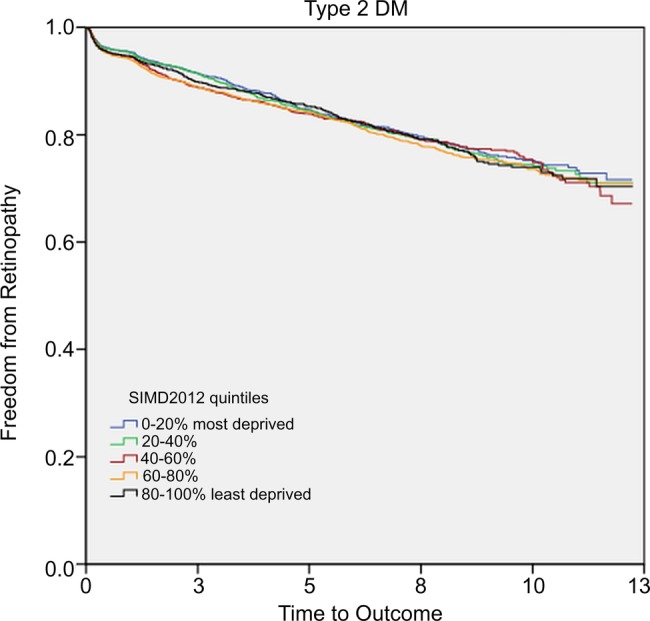
Kaplan-Meier survival curve of freedom from retinopathy in patients with type 2 DM. DM, diabetes mellitus; SIMD, Scottish Index of Multiple Deprivation.

There was no significant difference in the mean time to retinopathy in patients with type 2 DM from the different SIMD quintiles (p=0.427; figure 2; table 5).

**Table 5:** Retinopathy-free survival rates and HRs for type 2 DM according to the SIMD quintiles

		Survival (retinopathy-free)			
SIMD quintiles (%)	(N)	1 year (%)	5 years (%)	10 years (%)	HR (95% CI)*
0–20 most deprived	2896	95.4	84.7	75.2	0.95 (0.83 to 1.10)
20–40	2467	95.2	84.5	74.5	0.99 (0.86 to 1.15)
40–60	2556	94.4	83.8	75.4	1.03 (0.90 to 1.19)
60–80	4098	94.0	84.1	73.5	1.06 (0.93 to 1.20)
80–100 least deprived	2217	94.6	85.3	73.9	–

*ORs are derived from a Cox regression model, which also accounts for age and gender, and are relative to the most deprived SIMD quintile. SIMD quintile was not significant in this model (p=0.515).

DM, diabetes mellitus; SIMD, Scottish Index of Multiple Deprivation.

## Discussion

In our study, socioeconomic deprivation was associated with increased prevalence of DR in patients with type 1 DM, but not in patients with type 2 DM. This was independent of the duration of disease, HbA1c value, lipid profile and BP control. Patients with type 1 DM from a more deprived background were likely to develop retinopathy earlier than those from a less deprived background. Our finding of the association between longer duration of disease, higher HbA1c levels, higher BP, male gender and increased prevalence of DR is consistent with previous studies.^12–14^

### Strengths and weaknesses of the study

The strengths of our study lie in the large sample size and longitudinal cohort of patients who have been followed up systematically using the validated standardised yearly Scottish diabetic screening protocol. Through the integrated electronic patient record system, we were able to obtain measures of diabetic control, such as HbA1c levels, BP readings and cholesterol levels.^15^ However, one weakness of the study was the use of the most recent biochemistry and clinical data instead of the overall mean values. We were unable to attain information on the changes in postcode of the patients or account for mortality bias in this study.^16^ Another limitation to the study is the inherent bias in using screened populations. Patients who have not engaged with healthcare services or the screening programme would not have been included in the analyses. In addition, some patients would have presented with severe retinopathy and been treated under the care of the hospital and hence, were not included in the screening programme. This could result in selection bias if factors, such as socioeconomic deprivation, affect the likelihood of a patient being screened.

### Comparison with other studies

Previous studies have examined the influences of socioeconomic deprivation on DR in other parts of the UK. However, these studies did not account for other major risk factors such as duration of disease, HbA1c levels, lipid profile and BP control, all of which are known risk factors for development of DR. In the Gloucestershire cohort, socioeconomic deprivation was associated with sight-threatening DR, but not with non-sight-threatening DR.^17^ Similarly, in Avon and Somerset, patients with lower education levels were more likely to develop DR than those with higher education.^18^ However, in a Southampton cohort, Litwin *et al* found no association between relative affluence of residence and presence of retinopathy at time of diagnosis of type 2 DM.^19^

From a European perspective, the EURODIAB IDDM Complications study showed a higher prevalence of proliferative DR in men with lower educational qualifications compared with those with higher levels of education.^20^ In Spain, socioeconomic status was not independently associated with DR.^21^

Comparisons of development of retinopathy and socioeconomic deprivation between studies have to be carefully interpreted due to differences in measures of deprivation. Data compiled from self-reported questionnaires on income and educational attainment may have reporting biases.^18^ Moreover, most studies have only looked at baseline or cross-sectional screening outcomes and have not analysed these longitudinally over time.^22^ ^23^

### Meaning of the study

Eye healthcare in Scotland is provided free of charge, and with the use of mobile screening units, socioeconomic deprivation is not independently associated with increased prevalence of DR in type 2 DM. The lack of association between socioeconomic deprivation and DR in patients with type 2 DM in our study echoes the findings in a study conducted by Guthrie *et al*.^24^ They found that socioeconomic variations in the care of type 2 diabetes in Tayside have been largely eliminated under the pay-for-performance scheme (Quality and Outcomes Framework (QOF) 2004). However, younger patients were less likely to receive systematic care and have poorer control of intermediate outcomes. This is a reflection of complexity in the management of diabetes, which is beyond achieving ‘QOF targets’. Patient engagement and health behaviours play an important role. Indeed, the QOF was an effective instrument to incentivise care for older patients with type 2 DM, but its lack of efficacy in the control of DM in younger patients shows that it is not a ‘one-size-fits-all’ solution. This could be a possible explanation to the observation that SIMD has a significant relationship with prevalence of retinopathy in type 1 but not in type 2 DM.

It is worrying that despite free access to eye care in a well-developed diabetes care system, there is still an independent association between socioeconomic deprivation and increased prevalence of DR in type 1 DM. Whether socioeconomic deprivation is a cause or an effect of DR in patient with type 1 DM remains unclear. A possible explanation is that environmental factors within deprived areas may trigger immune responses in a genetically susceptible individual predisposed to type 1 DM and accelerate the development of complications.^25^ Another explanation could be a poorer control of diabetes in patients with lower socioeconomic background—we have previously shown that missed appointments in the diabetes retinal screening programme is associated with younger patients living in more deprived areas.^6^ On the other hand, the development of complications, such as retinopathy, may impact on the socioeconomic status of patients with type 1 DM. Poor diabetic control has been known to negatively affect academic achievement in school-aged children and reduce future career opportunities for patients with type 1 DM.^26^

Our study underscores the importance of targeted interventions for those patients most at risk of developing DR, in particular those with type 1 DM, and also the need for better allocation of resources to tackle inequality in eye healthcare. We need to find innovative ways to engage with our patients, especially with this younger age group, and support local health authorities in setting priorities for eye health initiatives to reduce inequalities.

### Unanswered questions and future research

Future studies should focus on elucidating the complex inter-relationship between socioeconomic deprivation, environmental influences and development of retinopathy in type 1 DM. Furthermore, we need to identify barriers to access health-seeking behaviours and unmet needs in patients from poorer backgrounds. Eye health equity profiles should be conducted within each locality, as these are the key to understanding inherent health inequalities within a distinct and unique community. This will, ultimately, help to improve the delivery of an effective multidisciplinary eye healthcare service.
